# Antineoplastic therapy affects the in vitro phenotype and functionality of healthy human bone marrow-derived mesenchymal stromal cells

**DOI:** 10.1007/s00204-024-03898-w

**Published:** 2024-11-12

**Authors:** Bo Scherer, Lucienne Bogun, Annemarie Koch, Paul Jäger, Uwe Maus, Laura Schmitt, Karina S. Krings, Sebastian Wesselborg, Rainer Haas, Thomas Schroeder, Stefanie Geyh

**Affiliations:** 1 Department of Hematology, Oncology and Clinical Immunology, Medical Faculty and University Hospital Duesseldorf, Heinrich Heine University Duesseldorf, Moorenstraße 5, 40225 Düsseldorf, Germany; 2 Department of Orthopedic Surgery and Traumatology, Medical Faculty and University Hospital Duesseldorf, Heinrich Heine University, Moorenstraße 5, 40225 Duesseldorf, Germany; 3 Institute for Molecular Medicine 1, Medical Faculty and University Hospital Duesseldorf, Heinrich Heine University, Universitätsstraße 1, 40225 Duesseldorf, Germany; 4 Department of Hematology and Stem Cell Transplantation, University Hospital Essen, Hufelandstraße 55, 45147 Essen, Germany

**Keywords:** MSC, Bone marrow microenvironment, Antineoplastic therapy, Hematotoxicity, Differentiation

## Abstract

While antineoplastic therapies aim to specifically target cancer cells, they may also exert adverse effects on healthy tissues, like healthy hematopoietic stem and progenitor cells (HSPC), leading to hematotoxicity as a common side effect. Mesenchymal stromal cells (MSC) are a major component of the bone marrow (BM) microenvironment, regulating normal hematopoiesis, while their susceptibility to anticancer therapies and contribution to therapy-related hematotoxicity remains largely unexplored. To address this, we investigated the effects of etoposide, temozolomide, 5-azacitidine, and venetoclax on healthy BM-derived MSC functionality. Doses below therapeutic effects of etoposide (0.1–0.25 µM) inhibited cellular growth and induced cellular senescence in healthy MSC, accompanied by an increased mRNA expression of *CDKN1A*, decreased trilineage differentiation capacity, and insufficient hematopoietic support. Pharmacological doses of 5-azacitidine (2.5 µM) shifted MSC differentiation capacity by inhibiting osteogenic capacity but enhancing the chondrogenic lineage, as demonstrated by histochemical staining and on mRNA level. At the highest clinically relevant dose, neither venetoclax (40 nM) nor temozolomide (100 µM) exerted any effects on MSC but clearly inhibited cellular growth of cancer cell lines and primary healthy HSPC, pointing to damage to hematopoietic cells as a major driver of hematotoxicity of these two compounds. Our findings show that besides HSPC, also MSC are sensitive to certain antineoplastic agents, resulting in molecular and functional alterations that may contribute to therapy-related myelosuppression. Understanding these interactions could be helpful for the development of strategies to preserve BM MSC functionality during different kinds of anticancer therapies.

## Introduction

Anticancer therapy, in particular cytotoxic chemotherapy, is often associated with adverse side effects on healthy tissues, resulting in increased morbidity and mortality (Crawford et al. 2008). Cytostatic drugs such as the topoisomerase II inhibitor etoposide (ETO) or the alkylating agent temozolomide (TMZ) target proliferating cells by intervening with the cell cycle, inducing DNA double-strand breaks, and eventually leading to apoptosis (Friedmann et al. 2000; Sinkule 1984). Other antineoplastic compounds, such as 5-azacitidine (AZA) or venetoclax (VEN), target cancer cells by interfering with epigenetic regulation and apoptosis mechanisms, respectively (Kaminskas et al. 2005; Scheffold et al. 2018). Hematotoxicity is a major side effect of most conventional and targeted therapies and has mainly been attributed to the effects of these drugs on cycling hematopoietic stem and progenitor cells (HSPC). Physiologically, HSPC reside within the bone marrow (BM) microenvironment. Mesenchymal stromal cells (MSC) are an integral component of the BM microenvironment and have an indispensable role in the regulation and support of HSPC by expression and secretion of regulatory factors, such as CXCL12, KITLG, or ANGPT1. Thereby, MSC are involved in orchestrating the balance of HSPC self-renewal and differentiation, helping to ensure the life-long supply of mature blood cells. In addition, MSC exhibit immunoregulatory properties and can differentiate into adipocytes, chondrocytes, and osteoblasts (Anthony and Link 2014; Mendez-Ferrer et al. 2010). Besides a direct effect of antineoplastic agents on HSPC, antineoplastic drugs may also exert effects on non-hematopoietic cells of the BM microenvironment, such as MSC, and thereby contribute to cancer therapy-related myelosuppression. So far, reports on the effects of conventional cytostatic drugs and novel therapies on MSC are relatively rare (Li et al. 2004; Qi et al. 2012; Rosca and Burlacu 2011; Ruhle et al. 2018), thus not allowing a definitive conclusion of their contribution to hematotoxicity. To address their potential role in therapy-related myelosuppression, we exposed healthy BM-derived MSC to conventional cytostatic drugs, as well as novel, more targeted therapies, and subsequently analyzed their phenotype and functionality.

## Materials and methods

### *Donor characteristics, cell isolation of MSC and CD34* + *cells and cell culture conditions*

BM samples were collected from 25 healthy donors (median age: 69.6 years, range 39–88 years) undergoing orthopedic surgery. This study was conducted in accordance with the ethical standards with the 1964 Helsinki Declaration and was approved by the ethics committee of the Heinrich Heine University, Düsseldorf (approval number 4777). All donors gave written informed consent.

MSC were derived from the mononuclear cell fraction as described previously (Geyh et al. 2013) and cultivated in Dulbecco´s Modified Eagle Medium (DMEM) low glucose with 25% FBS and 1% penicillin/streptomycin/L-glutamine (Sigma-Aldrich Chemie GmbH, St. Louis, MO, USA). All treatment experiments were performed in Passage 3–4. CD34 + cells were isolated from the mononuclear cell fraction of healthy donors using the Midi Magnetic Cell Separation (MACS) technology (Miltenyi, Bergisch Gladbach, Germany), cryopreserved, or directly cultured in Iscove’s Modified Dulbecco’s Medium (20% FBS and 1% penicillin/streptomycin/L-glutamine) supplemented with 10 ng/µL IL3, IL6, SCF, and 20 ng/µL FLT3L (all from Preprotech Inc., London, UK). Cell lines (THP-1, KG-1a, MV4-11, HL-60, K422, and SU-DHL-6) were purchased from the DSMZ (German collection of microorganisms and cell culture GmbH, Braunschweig, Germany) and cultivated according to the manufacturer’s instructions.

### Anticancer drug exposure

**Fig. 1:**
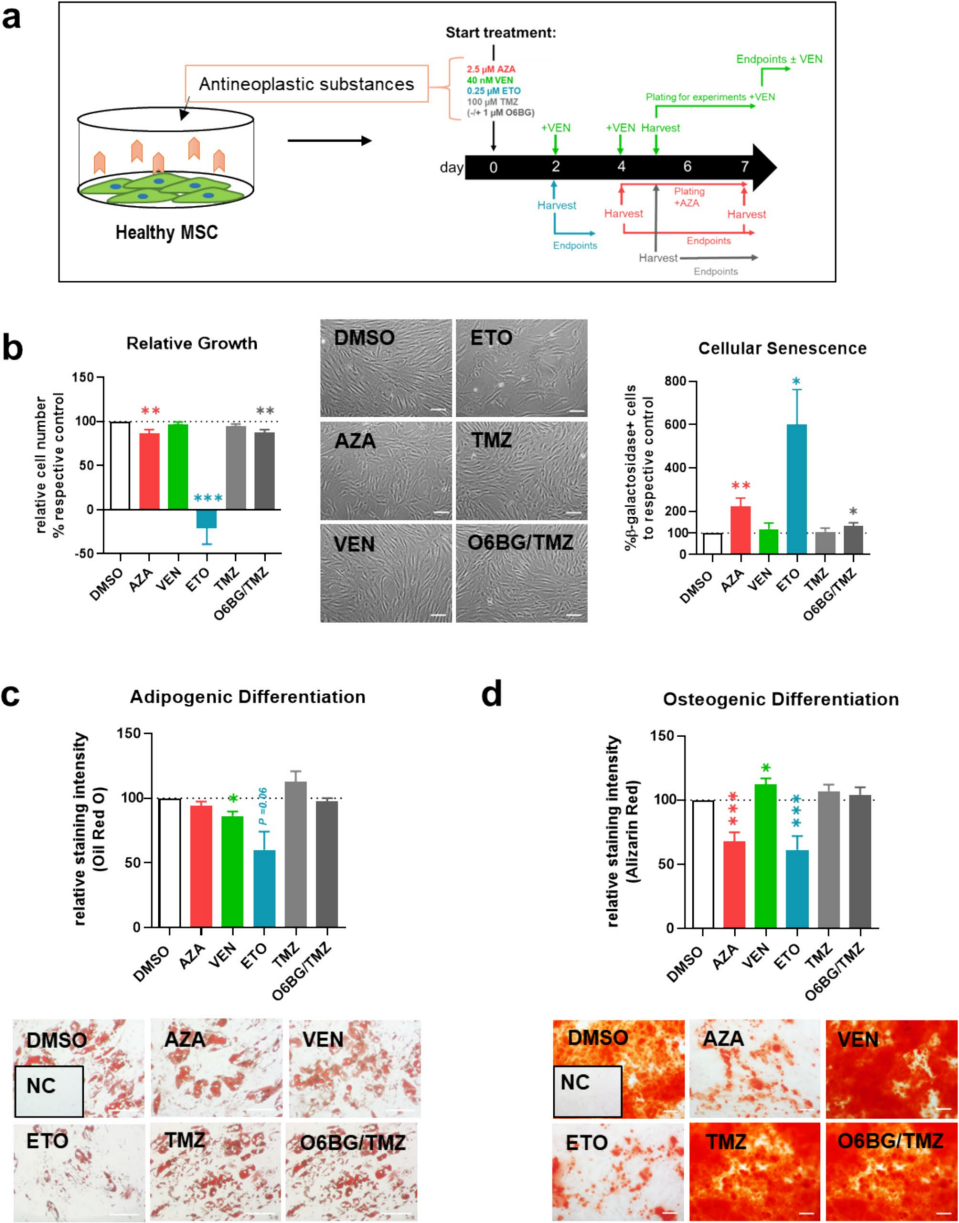
Phenotype, growth and differentiation of healthy, exposed MSC **a** Healthy MSC were exposed to antineoplastic agents or DMSO. Growth, cellular senescence and differentiation capacity were analyzed. **b** *Left side*: Relative cell numbers to the DMSO control were calculated and representative micrographs of MSC phenotype after exposure are given. *Right side*: Percentage of senescent cells as shown by β-galactosidase activity was calculated and normalized to the respective DMSO control. **c** Differentiation intensity of adipogenic induction of exposed healthy MSC was determined based on lipid vacuoles, stained with Oil Red O after 21 days. Representative micrographs are given. DMSO served as control and an un-induced negative control (NC) was included. **d** Alizarin Red staining intensity of exposed and osteogenic-induced MSC after 14 days are represented in comparison to the DMSO control. Bar charts display mean values and SEM. Statistical significance was determined by two-sided paired Student’s *t* test indicated by asterisks for at least four independent experiments *(*n ≥ 4; *P < 0.05 **P < 0.01 ***P < *0.001*). Scale bars indicate 100 µm

BM-derived MSC were plated at a density of 4 × 10^3^ cells per cm^2^, or in the case of ETO at a density of 6 × 10^3^ cells per cm^2^. Simultaneously, MSC were exposed to 2.5 µM AZA (#A2385, Sigma-Aldrich Chemie GmbH, St. Louis, MO, USA), 0.04 µM VEN (#S8048, Selleck Chemicals, Houston, USA), 0.1 and 0.25 µM ETO (#E1383, Sigma-Aldrich Chemie GmbH, St. Louis, MO, USA) or 100 µM TMZ (#T2577, Sigma-Aldrich Chemie GmbH, St. Louis, MO, USA), with and without pre-treatment with 1 µM O6-benzylguanine (O6BG; #73,762, STEMCELL technologies, Vancouver, Canada) to block the activity of reversal repair protein O6-methylguanine methyltransferase (MGMT). To mimic the clinical treatment duration, AZA treatment was continued until day seven, ETO was exposed for 48 h, and TMZ with or without O6BG and VEN were exposed for five days. VEN was repeatedly added every other day throughout the differentiation process. Details of the treatment scheme for MSC are depicted in Fig. 1A.

Primary CD34 + cells were treated with maximal doses of TMZ (with or without pre-treatment with 1 µM O6BG) and VEN at a density of 1.4 × 10^4^ cells per cm^2^ for five days. AML cell lines THP-1, KG-1a, MV4-11, and HL-60 were treated at a density of 5.2 × 10^4^ cells per cm^2^ for three days with TMZ and O6BG. Lymphoma cell lines SU-DHL-6 and K422 were treated at a density of 5.2 × 10^4^ cells per cm^2^ for five days with repeated exposure to VEN. Cell numbers were determined using the CASY® Cell Counter TTC (Roche, Basel, Schweiz) or by Trypan Blue staining.

### Analysis of cellular senescence by β-galactosidase staining

MSC were harvested after the respective treatment period and re-plated on a new culture plate. After 24 h, β-galactosidase activity was investigated using the Cellular Senescence Detection Kit (Merck KGaA, Darmstadt, Germany) following the manufacturer’s instructions. Stained cells were enumerated using the Axiovert 25 light microscope (Zeiss, Jena, Germany), and the fraction of senescent cells (β-galactosidase-positive) was assessed.

### Cell Cycle analysis and measurement of apoptosis

Cell cycle distribution and hypodiploid nuclei after exposure were determined using the Nicoletti assay (Nicoletti et al. 1991) by lysis of MSC with 100 µL of hypotonic buffer (1% sodium citrate, 0.1% Triton X-100) containing 50 µg/mL propidium iodide (#81845, Sigma-Aldrich Chemie GmbH, St. Louis, MO, USA). Analysis was performed by flow cytometry at the LSR-Fortessa™ and evaluated using BDFACSuite software (Becton Dickinson, Heidelberg, Germany).

### MSC differentiation

Trilineage differentiation of healthy MSC was initiated 24 h after treatment as previously described (Geyh et al. 2013). Adipogenesis was induced in DMEM high glucose medium (10% FBS and 1% penicillin/streptomycin/L-glutamine) with 0.1 mg/mL insulin, 0.1 µM dexamethasone, 0.2 mM indomethacin, and 1 mM IBMX, and cultivated with 0.01 mg/mL insulin for 21 days. Fat vacuoles were visualized with Oil Red O staining. Chondrogenesis was performed as 3D culture for 21 days in DMEM high glucose (1% penicillin/streptomycin/L-glutamine) supplemented with 1% ITS + 1, 1 µM dexamethasone, 50 µg/mL ascorbate-2-phosphate, 40 µg/mL L-proline, and 10 ng/mL TGFβ3 (all from Peptrotech, Inc., London, UK). Pellets were embedded in freezing medium and cryosectioned in 6 µm slices at the Leica Cryostat CM3050 (Leica, Wetzlar, Germany). Visualization of proteoglycan was performed by Safranin O staining. Osteogenesis was induced for 14 days with DMEM low glucose (25% FBS and 1% penicillin/streptomycin/L-glutamine) supplemented with 50 µg/mL ascorbic acid, 10 mM β-glycerol phosphate, and 0.1 µM dexamethasone. Calcium deposition was visualized by Alizarin Red staining. All reagents and supplements were obtained from SIGMA-Aldrich Chemie GmbH (St. Louis, MO, USA), unless otherwise stated. Images were captured at the Axiovert 25 microscope (Zeiss, Jena, Germany) and digitalized with the SPOT Software (Diagnostic Instruments Inc., Sterling Heights, MI, USA).

### Hematopoietic support analysis of MSC

MSC were plated at a density of 3 × 10^4^ per cm^2^ on tissue culture-treated 12-well plates and exposed to chemical agents. After indicated exposure times, medium was exchanged to agent-free medium. After 24 h, 1.5 × 10^4^ CD34 + cells per cm^2^ were added as direct co-culture for three days. Afterward, 1000 CD34 + cells were transferred to 1 mL methylcellulose containing erythropoietin (MethoCult™ H4434 Classic, STEMCELL technologies, Vancouver, Canada), plated in duplets and cultivated at 37 °C and 5% CO_2_ for 14 days for colony-forming units (CFU) assay. Colonies were enumerated using the Axiovert 25 microscope (Zeiss, Jena, Germany) and differentiated between white (CFU-GM, CFU-G, CFU-M), red (CFU-E, BFU-E) and mixed (CFU-GEMM) colonies as previously described (Jager et al. 2021).

### Quantitative real-time PCR (qPCR)

RNA was purified directly after treatment exposure using the RNase Micro or Mini Kit (QIAGEN, Hilden, Germany) including optional DNase digestion. Transcription to cDNA was performed by Superscript II TM Reverse transcriptase (Invitrogen, Darmstadt, Germany). qPCR was performed in duplicates on a StepOne Plus Real-time PCR Cycler or QuantStudio^TM^3 System using SYBR Green PCR Master Mix (all from Applied Biosystem, Life Technologies, Carlsbad, CA, USA). Primer sequences can be provided on request. *GAPDH* served as reference control. Differences in mRNA expression levels were calculated as fold change by the ∆∆CT method.

### Statistical analysis

Statistical analysis was performed using Prism 8.4.3 (GraphPad Software Inc., La Jolla, USA) using the two-sided paired Student’s t test for parametric data, or Wilcoxon matched-pairs signed-rank test of non-parametric data. Data graphs show mean and SEM unless otherwise stated. Significance was determined at *P* < *0.05* and is indicated by asterisks (*P ≤ 0.05, **P ≤ 0.01, ***P ≤ *0.001*).

## Results

### Exposure to antineoplastic agents affect phenotype, growth and differentiation capacity of healthy MSC

In vitro cultured healthy MSC are usually characterized by a spindle-like phenotype, plastic adherence and trilineage differentiation capacity. MSC were cultivated in the presence of clinical doses of 5-azacitidine (AZA), venetoclax (VEN), etoposide (ETO), or temozolomide (TMZ) alone or co-treated with the MGMT inhibitor O6-benzylguaine (O6BG/TMZ) following the scheme depicted in Fig. 1a. Neither VEN nor TMZ induced phenotypical alterations in MSC or altered growth behavior or differentiation capacity (Fig. 1b-d). Slight phenotypical and proliferative alterations of MSC were found after AZA with decreased cumulative population doublings (CPD) by 13% (*P* < *0.0001*) and a roughly doubled cellular senescence (*P* < *0.01*). Functionally, AZA exposure inhibited osteogenic differentiation in healthy MSC as shown by decreased Alizarin Red staining (*P* < *0.001),* but not adipogenesis*.* After ETO exposure, MSC displayed a broad and disorganized morphology and the proliferative activity of MSC diminished completely after 48 h exposure (mean population doublings (PD), DMSO: 1.0, ETO: -0.2, *P* < *0.001)* accompanied by a sixfold increase in β-galactosidase-positive cells (*P* < *0.05*), indicating cellular senescence. Differentiation into adipocytes and osteoblasts was substantially inhibited following ETO exposure (Fig. 1b-d).

### Etoposide induces irreversible cell cycle arrest in MSC

**Fig. 2:**
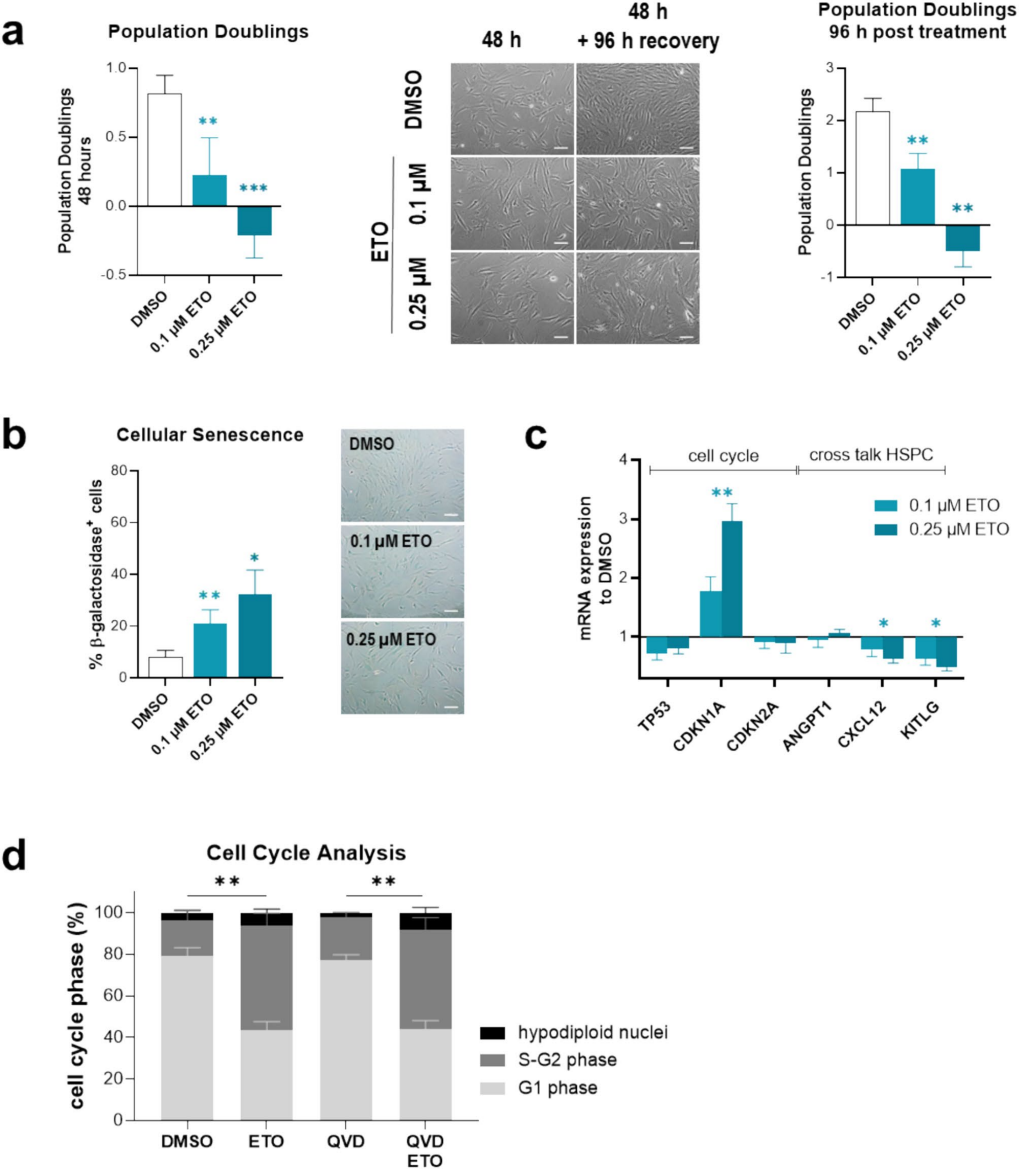
Cellular senescence and cell cycle arrest after exposure to ETO Healthy MSC were exposed to 0.1 or 0.25 µM ETO for 48 h. **a** *Left side*: PD were calculated after 48 h of exposure to 0.1 µM and 0.25 µM ETO. DMSO served as solvent control. *Middle*: representative micrographs of MSC phenotype after 48 h exposure or additional 96 h recovery period. Scale bars indicate 100 µm. *Right side*: PD of exposed MSC with a recovery period of 96 h. **b** Percentage of β-galactosidase-positive cells after exposure to 0.1 µM and 0.25 µM ETO. **c** Exposed MSC were analyzed by qPCR for cell cycle marker *TP53*, *CDKN1A*, *CDKN2A* on mRNA level as well as for microenvironment regulatory factors *ANGPT1*, *CXCL12* and *KITLG*. Dose-dependent dysregulation of *CDKN1A*, *CXCL12* and *KITLG* was determined. **d** Flow cytometry of Nicoletti staining revealed absence of apoptosis in ETO-exposed MSC, but showed a clear shift of cell cycle stage to S–G_2_. Mean values and SEM are shown for at least three independent experiments (n ≥ 3). Asterisks indicate statistical significance using Student’s t test vs DMSO (*P < *0.05*, **P < *0.01*, ***P < *0.0001)*

ETO prominently affected phenotype and growth of healthy MSC after 48 h of exposure already at sub-clinical concentrations. Therefore, we tested an even lower concentration of 0.1 µM ETO and found similar effects regarding phenotypical alterations and decreased PD in a dose-dependent manner, which were not recovered after four days without exposure (Fig. 2a; mean PD recovery, DMSO: 2.2, 0.1 µM ETO: 1.1, 0.25 µM ETO: − 0.5, *P* < *0.01*). Similarly, increased cellular senescence and selective induction of cell cycle marker *CDKN1A* were already determined at 0.1 µM and more prominent at the higher dose. Downregulation of hematopoiesis-relevant factors *CXCL12* and *KITLG* after two days of culture was determined (Fig. 2b, c). Cell cycle arrest in the absence of apoptosis was shown by Nicoletti assay with the majority of ETO-exposed MSC remaining in S–G_2_ phase (mean proportion of cells in S–G_2_, DMSO: 17.3%, 0.25 µM ETO: 50%) (Fig. 2d).

### 5-Azacitidine inhibits osteogenic potential, but enhances chondrogenic differentiation capacity in healthy MSC

**Fig. 3:**
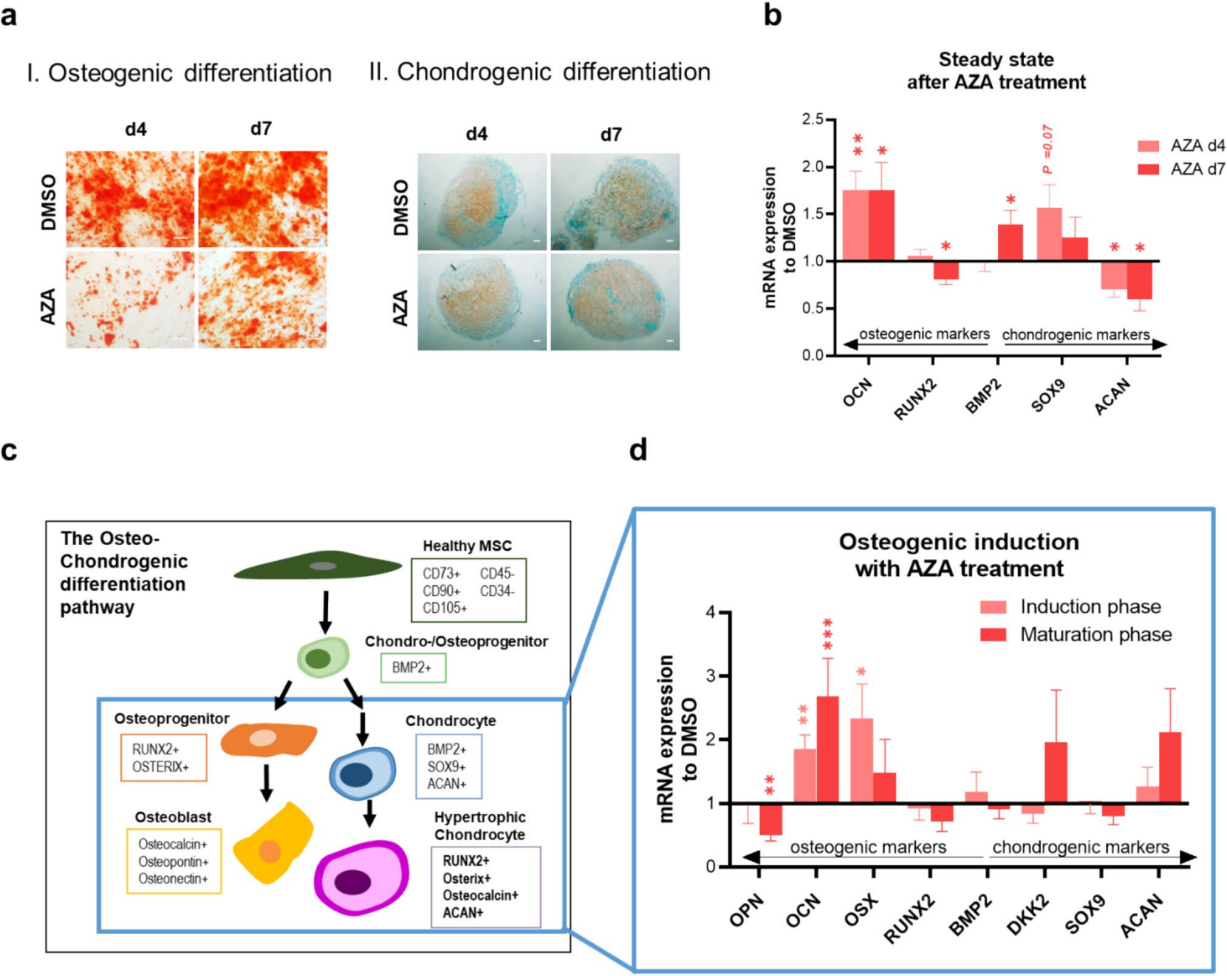
5-Azacitidine induces differentiation shift from osteogenic to preferentially chondrogenic differentiation **a** MSC were exposed to AZA for four and seven days and afterward induced for osteogenic or chondrogenic differentiation. AZA exposure led to inhibition of calcium disposition after osteogenic induction for 14 days as shown by Alizarin Red staining, while proteoglycan was robustly incorporated during chondrogenic induction as visualized by Safranin O staining after 21 days of differentiation. Representative images are shown. Scale bars indicate 100 µm. **b** Steady-state transcriptional regulation after AZA exposure showed upregulation of osteo-chondrogenic factor *bone morphogenetic protein-2* (*BMP2)*, but differential regulation of specific osteogenic and chondrogenic markers. **c** Schematic depiction of the osteo-chondrogenic differentiation pathway and involved regulating factors. Graphics were generated using Microsoft PowerPoint 2013. **d** qPCR results of mRNA expression levels of osteogenic–chondrogenic factors (*BMP2, RUNX2, DKK2, OSX, OCN, SOX9, ACAN*) during 14 days of osteogenic induction and simultaneous AZA treatment. Fold changes of the respective expression related to the control group (normalized as 1) are given for induction phase (days 1–7) and maturation phase (days 10–14) of osteogenic differentiation. Mean values and SEM are shown for at least four independent experiments (n ≥ 4). Asterisks indicate statistical significance using Student’s t test (*P < *0.05*, **P < *0.01*, ***P < *0.0001)*

We found that AZA clearly inhibited osteogenic potential of healthy MSC (Fig. 1d). Knowing that osteoblasts share a common progenitor with chondroblasts and osteogenic–chondrogenic lineage factors, alterations in chondrogenic differentiation were assumed. Therefore, AZA-exposed MSC were differentiated into the chondrogenic lineage. However, Safranin O staining of chondrogenic differentiated cell pellets revealed no differences to the control group, but even showed a tendency to a more robust and concentrated proteoglycan disposition (Fig. 3a, II). To investigate differentiation regulation after AZA exposure on the molecular level, mRNA expression of early and late osteogenic and chondrogenic markers in exposed, but still un-differentiated MSC, was determined (Fig. 3b). Interestingly, the late osteogenic marker Osteocalcin (*OCN*) was clearly upregulated, while not stained by Alizarin Red in osteoblastic-differentiated MSC after AZA (Fig. 3a, I) On the other hand, early osteogenic marker *RUNX2* was downregulated, while early chondrogenic marker *SOX9* was upregulated. However, *ACAN*, a late marker of chondrogenesis, was downregulated. To elucidate this diverse dysregulation of differentiation markers in MSC after AZA, we more closely investigated the regulation of common markers on mRNA level during the course of osteogenic differentiation (induction phase (days 1–7) and maturation phase (days 10–14). Strikingly, we found upregulation of early and late osteogenic markers after AZA exposure. However, at the same time, Dickkopf-2 (*DKK2)*, a WNT regulator and inhibitor of osteogenesis, and late chondrogenic marker aggrecan (*ACAN*) were upregulated as well (Fig. 3d). These findings, together with an increased expression of early chondrogenic inducer *SOX9* in un-differentiated AZA-exposed MSC, as well as increased *OCN* levels after AZA treatment, suggest that AZA may skew the differentiation of MSC toward the chondrogenic lineage, at the expense of the osteoblastic lineage.

### Temozolomide and venetoclax specifically target malignant cells, but do not affect healthy MSC

**Fig. 4:**
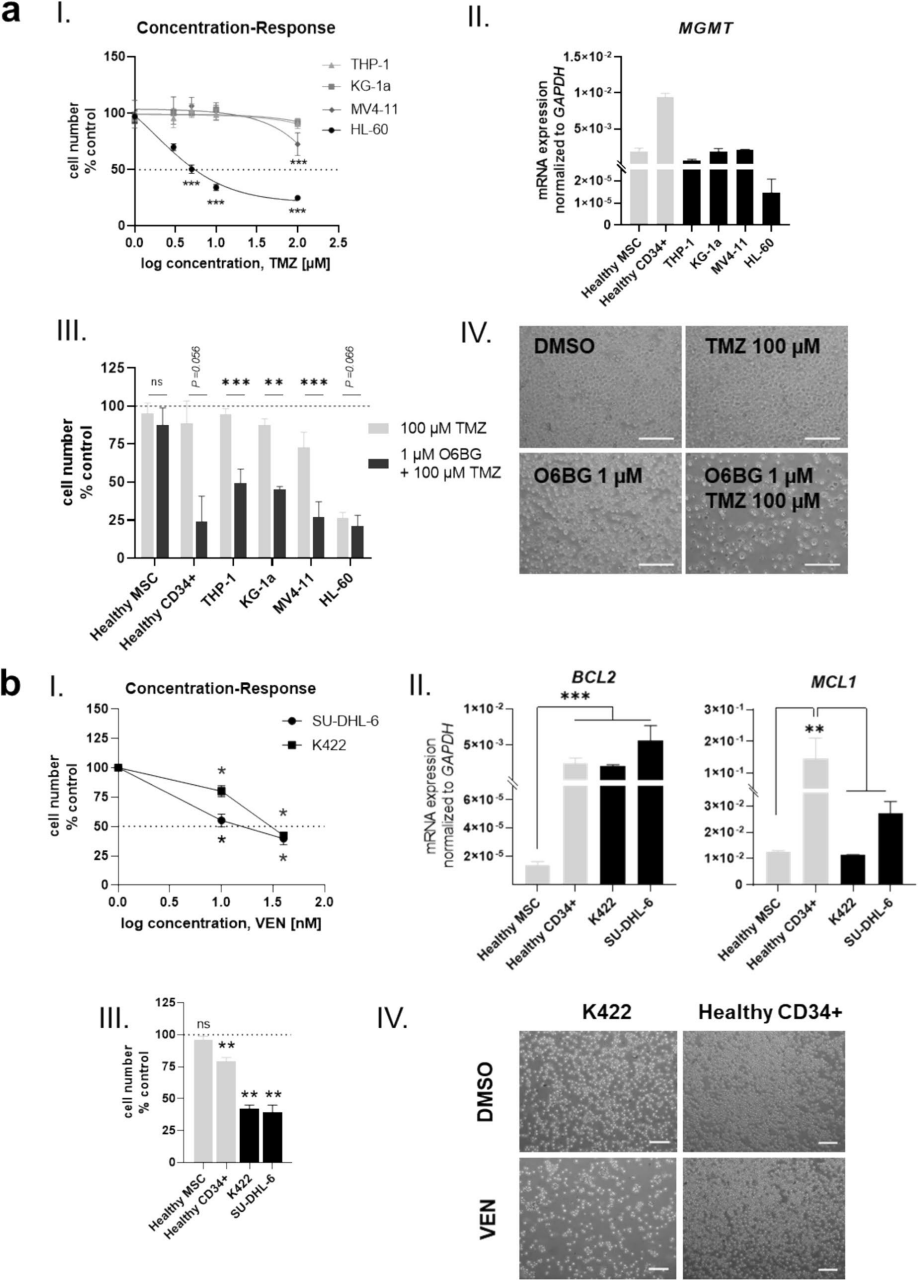
Temozolomide and venetoclax target hematopoietic cells, but not healthy MSC **a** I: Concentration response curve of AML cell lines THP-1, KG-1a, MV4-11 and HL-60 after three days exposure to increasing concentrations from 1 to 100 µM of alkylating TMZ are shown. II: qPCR of *MGMT* mRNA expression in native MSC, CD34 + cells and AML cell lines. III: Relative cell growth after co-treatment of AML cell lines and healthy MSC and CD34 + cells with TMZ and O6BG. IV: Representative images of AML cell line MV4-11 with TMZ with or without MGMT inhibitor O6BG are shown. DMSO served as control. **b** I: Concentration response of lymphoma cell lines SU-DHL6 and K422 to VEN after seven days of culture. II: qPCR of *BCL2* and *MCL1* mRNA expression in native MSC, CD34 + and lymphoma cell lines SU-DHL6 and K422. III: Cell lines, MSC and CD34 + were exposed to VEN for 5 days and subsequently cell numbers were determined. IV: Representative micrographs of lymphoma cell line K422 and healthy CD34 + cells after exposure to VEN for five days are shown. DMSO served as control. Scale bars indicate 100 µm. Mean values and SEM are shown for at least three independent experiments (n ≥ 3). Asterisks indicate statistical significance using Student’s t test vs DMSO (*P < *0.05*, **P < *0.01*, ***P < *0.0001)*

Having observed no effects on the growth or functionality of healthy exposed MSC after maximal doses of TMZ and VEN, we investigated the efficacy of TMZ and VEN on AML and lymphoma cell lines respectively. For this purpose, four AML cell lines (HL-60, THP-1, KG-1a, MV4-11) were exposed to increasing concentrations ranging from 1 µM up to 100 µM of TMZ. THP-1 and KG-1a showed no response to TMZ, while a decreased cell number of MV4-11 was observed at the highest concentration of TMZ. HL-60 cell numbers decreased in a dose-dependent manner, already at the lowest TMZ concentration (Fig. 4a, I). Sensitivity of AML cell lines toward TMZ concentration correlated with the mRNA expression of the DNA damage repair enzyme *MGMT* with the sensitive cell line HL-60 exhibiting low mRNA levels of *MGMT* compared to other AML cell lines. In agreement with this, healthy MSC showed mRNA expression levels of *MGMT* comparable to resistant AML cell lines, while healthy CD34 + HSPC exhibited a 4.5-fold higher mRNA expression level of *MGMT* than AML cell lines (Fig. 3a, II). Co-treatment of TMZ and MGMT inhibitor O6BG showed significantly diminished cell numbers of all AML cell lines, as well as healthy CD34 + cells. MSC were not sensitive to the combination of TMZ with O6BG (Fig. 4a, III).

Regarding VEN, two lymphoma cell lines (SU-DHL-6, K422) were exposed to clinically relevant doses ranging from 10 to 40 nM and showed dose-dependent decrease in cell number, with SU-DHL-6 being more sensitive (Fig. 4b, I). On mRNA level, *BCL2* expression was similar in healthy CD34 + cells, K422 and SU-DHL-6, while healthy MSC exhibited low *BCL2* expression. On the other hand, *MCL1* expression was significantly higher in healthy CD34 + cells compared to healthy MSC and lymphoma cell lines (Fig. 4b, II). Accordingly, exposure of 40 nM VEN led to significantly diminished cell numbers in both lymphoma cell lines*,* while healthy MSC and CD34 + HSPC were not as drastically affected (Fig. 4b, III, IV).

### Antineoplastic agents affect hematopoietic support capacity of healthy MSC

**Fig. 5:**
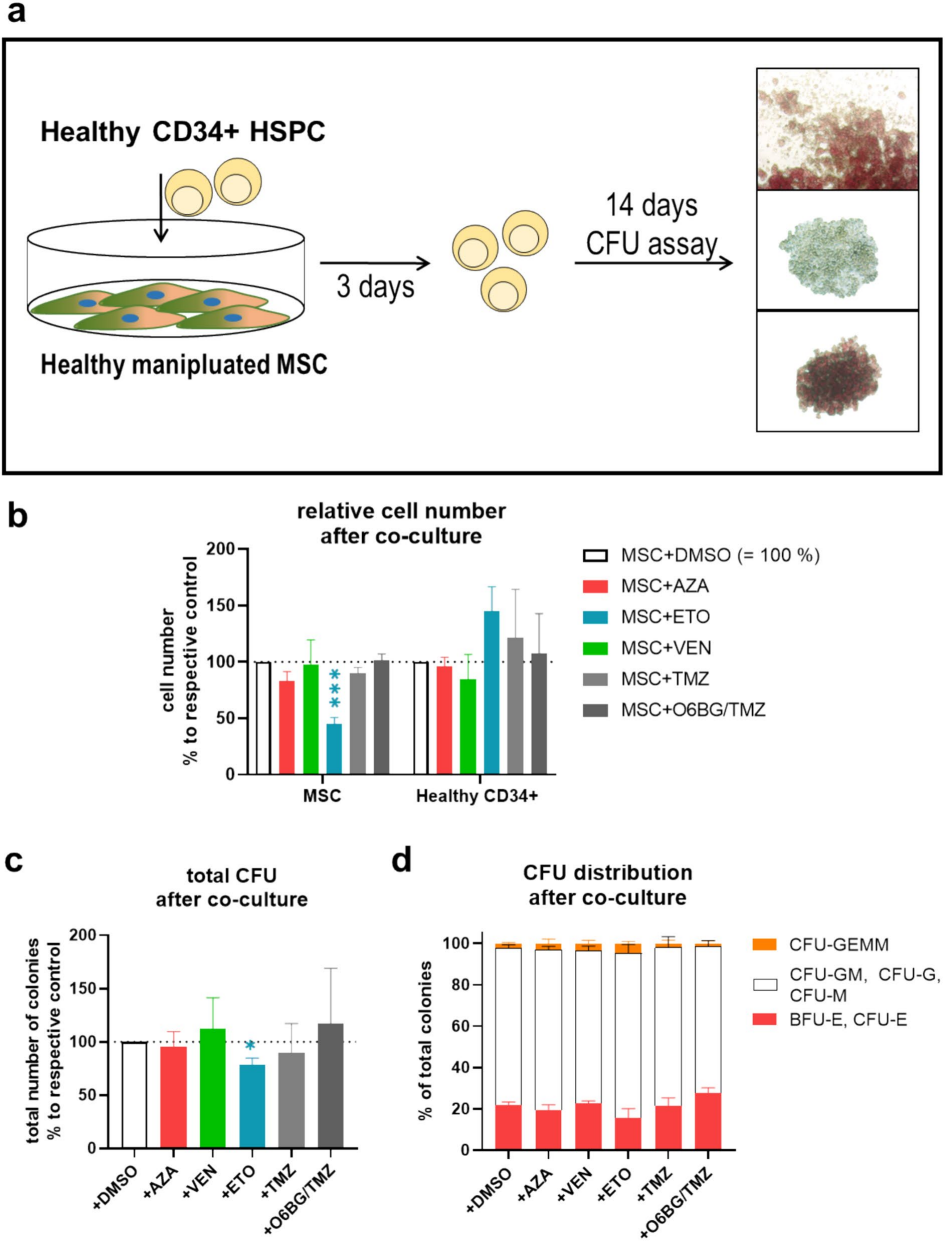
Hematopoietic support capacity of exposed MSC **a** Study design: MSC were pre-treated for 24 h with AZA or ETO, or six days with VEN and TMZ (± O6BG) and subsequently co-cultured for three days with healthy CD34 + cells in medium without antineoplastic agents. Afterward, CFU assay was performed for 14 days with co-cultured CD34 + HSPC to investigate their differentiation potential toward CFU-GEMM, BFU-E, CFU-E, CFU-G, CFU-M, CFU-GM. Colonies were enumerated under a light microscope. **b** Cell numbers of MSC and CD34 + were determined after three days of co-culture. **c** Total number of colonies of co-cultured CD34 + cells after 14 days of CFU assay. **d** CFU-GEMM, BFU-E, CFU-E, CFU-G, CFU-M, CFU-GM MSC were counted and are depicted in respective colors. CD34 + cells co-cultured with MSC supplemented with DMSO served as control. Mean values and SEM are shown for at least three independent experiments (n ≥ 3). Asterisks indicate statistical significance using Student’s t test vs DMSO (*P < 0.05; ***P < *0.001*)

Given the pivotal role for hematopoiesis under physiological circumstances, which are mediated by secreted factors as well as ligand–receptor interactions, we investigated the hematopoietic support capacity of MSC after exposure to antineoplastic agents next. For this purpose, healthy CD34 + cells were co-cultivated with MSC, which were previously exposed to the cytostatic drugs, and subsequently subjected to CFU assays (Fig. 5a). After three days of co-culture with exposed MSC, relative cell numbers of MSC were comparable to findings in Fig. 1b. CD34 + cell numbers were not altered by antineoplastic agents, however showed a clear tendency to an increased cell number after co-culture with ETO-exposed MSC (Fig. 5b). Regarding CFU assays, only MSC previously exposed to ETO showed a significantly diminished hematopoietic support capacity as indicated by a lower number of total colonies, while the distribution along the different colony subtypes was not altered significantly (Fig. 5c, d).

## Discussion

The majority of anticancer therapies is associated with an impairment of healthy hematopoietic progenitor cells, leading to dose-limiting hematotoxicity or the development of therapy-related neoplasms (Bertrums et al. 2022; Diamond et al. 2023; McNerney et al. 2017). So far, the main focus of chemotherapy-induced hematotoxicity has been on HSPC with elaborate research on cytoprotective strategies for HSPC to mitigate myelosuppressive effects (Fakhrabadi et al. 2020; List et al. 1996; Sawai et al. 2001). Despite the regulatory role of MSC in the hematopoietic system, the impact of different anticancer therapies on MSC and specifically their contribution to hematotoxicity remain incompletely understood. Therefore, we investigated this using human BM-derived MSC from healthy donors with a corresponding age of cancer patients for clinical relevance. It has to be noted that age-related clonal hematopoiesis and accompanying bone marrow alterations were not tested in this cohort and therefore the significance of clonal hematopoiesis regarding MSC sensitivity toward anticancer therapy cannot be derived here (Hecker et al. 2021; Winter et al. 2024). Nevertheless, our comprehensive *in vitro* analyses show that MSC are directly affected by some antineoplastic agents (ETO, AZA) and thereby contribute to hematotoxicity, demonstrating the relevance of MSC and their therapeutic response for further investigations.

ETO induced cellular senescence in MSC already at concentrations below clinical plasma levels of 0.1–0.25 µM, which was not reversible, neither in proliferative medium nor by induction of differentiation. Nicoletti assay revealed efficient cell cycle inhibition by ETO in the absence of apoptosis. Similar findings were reported by other groups, showing that MSC are largely resistant to apoptosis but undergo cellular changes, e.g., cellular senescence (Lutzkendorf et al. 2017; Nicolay et al. 2016; Qi et al. 2012). Increased proportion of β-galactosidase-positive cells, accompanied by the induction of senescent-associated markers as shown here, suggests the induction of the senescence-associated secretory phenotype (SASP). This phenotype is pro-inflammatory and shown to lower the viability and differentiation capacity of HSPC (Demaria et al. 2017; Tchkonia et al. 2013). Although we did not analyze the SASP of ETO-exposed MSC, together with our findings, this may explain the insufficient hematopoietic support of MSC exposed to topoisomerase II inhibitors. Within a clinical setting, the application of senolytics to manage therapy-induced senescence might help preserve a healthy microenvironment during treatment (Kirkland and Tchkonia 2020), a view that is supported by studies in mice showing that senolytics enhance MSC differentiation capacity (Zhou et al. 2021). Hence, the modulation of MSC by senolytics or molecular manipulation, e.g., by retroviral transfection, may help maintain the niche equilibrium and support healthy hematopoiesis (Halim et al. 2020; Sawai et al. 2001).

On the other hand, AZA led to a differentiation shift of MSC, clearly inhibiting osteogenic differentiation of healthy MSC and favoring differentiation toward the chondrogenic and adipogenic lineage. As hypomethylating agent, AZA was reported to reverse the hypermethylation patterns in MDS stroma and affect epigentically regulated differentiation processes (Bhagat et al. 2017). However, described effects of AZA on the differentiation capacity of MSC are controversial and range from enhanced potential (Bae et al. 2017; Yan et al. 2014) to decreased potential in individual lineages of differentiation (Rosca and Burlacu 2011; Wenk et al. 2018) and are most likely dependent on the cellular origin and treatment scheme. Clinically, patients are exposed to 75 mg/m^2^ body surface with a bioavailability of around 89% (Derissen et al. 2014; Kaminskas et al. 2005). In this study, we have chosen a low concentration (2.5 µM) within patient plasma levels, which can range from 1 to 50 µM, and mimicked the treatment duration of seven days (Kaminskas et al. 2005). Moreover, we used cells derived directly from human BM, while other studies were conducted in mice (Rosca and Burlacu 2011) or with MSC derived from adipose tissue (Yan et al. 2014). Although we did not observe effects on the hematopoietic support function, the essence of osteoblasts in hematopoietic homing is well known (Neiva et al. 2005) and might implicate the initiation of a dysbalance in niche equilibrium, potentially indirectly affecting HSPC crosstalk and regulation (Raaijmakers et al. 2010). Due to the epigenetic mode of action of AZA, dose escalation might reveal differential effects. Wenk and colleagues (2018) used a fourfold higher concentration with a lower exposure time (48 h) and found osteogenic-enhancing effects on healthy MSC and MSC from patients with MDS, while adipogenic differentiation was inhibited. In this set-up, the hematopoietic support function was increased as well, demonstrating the importance of osteogenic potential on the one hand and dose selection on the other.

In our analysis, TMZ and VEN clearly targeted hematopoietic cells but not MSC. We did not find notable alterations in MSC at the highest clinically relevant dose of 100 µM TMZ (Meany et al. 2009; Ostermann et al. 2004) or 40 nM VEN (Scheffold et al. 2018). On the one hand, TMZ effectively targeted malignant cells, as reflected by decreased growth after exposure, which was dependent on *MGMT* expression level. The overall response of cancer patients treated with TMZ is dependent on their MGMT activity, with patients conferring high activity being resistant to TMZ treatment (Marrari et al. 2011; Trillo Aliaga et al. 2021). Resistant cells usually become sensitized by MGMT inhibition, as shown here and by others (Chen et al. 2022), but this does not apply to healthy MSC in our experiments. The slow proliferative activity of MSC compared to cancer cell lines must be noted; however, TMZ exerts its effects after two cell cycles according to its mode of action, which was given in each tested cell population. Studies have shown that myelosuppression by TMZ correlates with *MGMT* expression in leukocytes (Stokes et al. 2012). On the other hand, primitive CD34 + HSPC were exposed to TMZ and showed clear sensitivity when exposed in combination with the MGMT inhibitor. This points to the fact that the origin of TMZ-induced myelosuppression is probably also caused by the suppression of HSPC. Although HSPC already present with the relative highest *MGMT* expression in our cohort, alkylating agents are often combined with O6BG to potentiate TMZ effects, thereby also targeting HSPC. To protect HSPC reservoirs, MGMT expression and activity could be artificially increased in these cells to ensure selective elimination of cancer cells (Pollok 2003; Reese et al. 1999).

Similarly, VEN specifically inhibits the anti-apoptotic protein BCL2, which is mainly increased in cancer cells. Mainly hematological neoplasms, such as acute myeloid leukemia or lymphomas are treated with VEN as part of a combination therapy. In lymphomas, for example, the common chromosomal translocation t(14;18)(q32;q21) leads to a constitutive overexpression of BCL2, allowing apoptotic escape for malignant cells (Miyashita and Reed 1993; Weiss et al. 1987). Therefore, VEN selectively targets these cells and induces apoptosis. Compared to cancer cell lines and HSPC, the expression of *BCL2* is much lower in healthy MSC, explaining their lack of alterations after VEN exposure. In fact, CD34 + cells have similar levels of *BCL2* to lymphoma cell lines due to their immaturity (Delia et al. 1992) but were not as dramatically affected by VEN. Following this, we detected a tenfold increased expression of the alternative anti-apoptotic *MCL1* in healthy CD34 + cells compared to lymphoma cell lines, protecting the progenitor cells from long-lasting abolishment of BCL2 by VEN. Consequently, only cells conferring both, high *BCL2* and low *MCL1* levels are specifically and durably targeted by VEN (Warren et al. 2019). While it was shown that VEN spares hematopoietic cells in peripheral blood (Chen et al. 2020; Shi et al. 2021; Souers et al. 2013), knowledge about alterations in hematopoietic progenitors and their potential contribution to myelosuppression does not exist. To determine the etiology of VEN-related myelosuppression, functional effects on healthy CD34 + HSPC should be investigated in future.

## Conclusion

The new insights into direct MSC alterations provided by this study emphasize the critical need to consider the entire BM microenvironment in the context of anticancer therapies. While not all antineoplastic agents mediate their myelosuppressive effects via BM stromal cells, substance-specific effects directly affecting MSC were found after ETO and AZA exposure, potentially contributing to therapy-related hematotoxicity. These results open a new perspective on the management of myelosuppressive side effects during anticancer therapy and should be further investigated for developing novel strategies implementing the relevance of BM MSC.

## Data Availability

The data that support the findings of this study are available from the corresponding author upon reasonable request.
